# Mechanisms of Chiral Induction to Foldamer Backbones

**DOI:** 10.1002/chem.202502433

**Published:** 2025-10-08

**Authors:** Govinda Prasad Devkota, Roshan Lama, C. Scott Hartley

**Affiliations:** ^1^ Department of Chemistry and Biochemistry Miami University Oxford Ohio USA

**Keywords:** computational chemistry, conformational analysis, foldamers, helicity, stereochemistry

## Abstract

Foldamers, oligomers that adopt well‐defined conformations, represent an efficient strategy toward nanoscale structural complexity. While most foldamers fold into helices, many abiotic foldamers are built from achiral repeat units and therefore do not have a preferred twist sense. Their handedness can, however, be controlled by attaching groups with chirality centers to the foldamer backbone. This process allows chiral information from readily available compounds to be amplified into larger‐scale structural asymmetry and translated into functional behavior. This review describes mechanisms whereby the point chirality of chiral “controller” groups directs foldamer twist sense. We highlight examples of aromatic oligoamides, oligohydrazides, oligoindoles, oligo(*ortho*‐phenylenes), oligooxymethylenes, and oligo(aminoisobutyric acids), examining cases where the controller groups are attached at either the helices’ termini or sides. Our emphasis is on applying intuitive concepts from conformational analysis and, where appropriate, computational modeling of small substructures. In each case, we consider first short‐range interactions that orient the controller group relative to its direct point of attachment to the foldamer, and then its long‐range interactions with more‐distant parts of the oligomer. Together, these interactions allow the twist sense to be predicted (or at least rationalized). Understanding these mechanisms should facilitate the design of systems with dynamic control over helicity.

## Graphical abstract

Helical foldamers are often built from achiral components and thus have no inherent bias to their twist sense. This review describes mechanisms for controlling their handedness using appended stereocenters. It examines how point chirality directs the twist sense through a combination of short‐range interactions to the directly attached repeat unit and long‐range interactions with groups farther along the backbone.

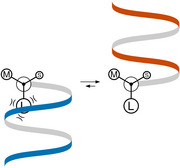

## 1 Introduction

Nature has developed complex strategies for folding biomacromolecules, such as proteins and nucleic acids, into the complex 3D structures essential for their function. These natural systems serve as the inspiration for artificial systems known as “foldamers”, synthetic oligomers that adopt well‐defined conformations.^[^ ^1^, ^2^ ^]^ Their folding is usually controlled using noncovalent interactions, including hydrogen bonding, arene–arene stacking, van der Waals forces, electrostatic interactions, and solvophobicity. Foldamers allow the efficient generation of nanoscale structural complexity, making them attractive candidates for applications in molecular recognition,^[^ ^3^, ^4^, ^5^, ^6^, ^7^ ^]^ catalysis,^[^ ^8^, ^9^ ^]^ electronics,^[^ ^10^, ^11^, ^12^ ^]^ self‐assembly,^[^ ^13^, ^14^ ^]^ biology,^[^ ^15^, ^16^ ^]^ and materials.^[^ ^17^ ^]^

The most common secondary structure in abiotic foldamers is the helix, which is chiral. Unlike biomolecules, many synthetic foldamers are constructed from achiral repeat units; consequently, a helical fold will have no inherent handedness preference. Instead, the native state for many abiotic foldamer backbones is a dynamic racemate of right‐ (*P*) and left‐handed (*M*) helices. Biasing this equilibrium has significant implications, particularly for applications that require the foldamer to interact with other chiral species. Further, the issue of chiral induction in foldamers—that is, favoring one twist sense by appending a chiral group—is representative of the general problem of relaying structural information throughout a larger (super)molecule. This issue is important to understanding how the geometry changes of molecular‐level devices, such as switches and motors,^[^ ^18^, ^19^, ^20^, ^21^ ^]^ can be amplified into larger motions.

There are two primary strategies for inducing a preferred twist sense in helical foldamers composed of achiral monomers: (1) the binding of chiral guests^[^ ^22^, ^23^, ^24^, ^25^ ^]^ and (2) the attachment of chiral moieties either at the backbone,^[^ ^26^, ^27^, ^28^ ^]^ termini,^[^ ^29^, ^30^, ^31^, ^32^, ^33^, ^34^ ^]^ or side‐chains.^[^ ^35^, ^36^, ^37^, ^38^ ^]^ In both cases, the combination of the helix with another chiral element gives diastereomeric twist senses, which are thus formed in unequal ratio. In this review we focus on the second case. Using covalently attached stereogenic units to direct folding is simpler as a strategy to engineer molecular geometry and conceptually closer to the biomolecular inspiration for foldamers (in the sense that peptides and nucleic acids are inherently asymmetric). This approach also leaves the helix free to interact with other species for molecular recognition, an important application. The attached chiral moieties, which we will call “controller groups”, usually incorporate chirality centers. The groups attached to the chirality center we will refer to as its ligands.^[^ ^39^ ^]^

This review does not discuss helical polymers. While they exhibit fascinating stereochemical behavior (e.g., Green's sergeant‐and‐soldiers model), they have been reviewed extensively elsewhere.^[^ ^40^, ^41^, ^42^ ^]^ We also do not discuss foldamers with stereogenic units incorporated directly into their backbones (e.g., most peptides and related species^[^ ^43^, ^44^, ^45^, ^46^ ^]^). Our focus is how point chirality from one appended controller group can be translated into twist sense excess in otherwise unbiased helices. This review is also not intended to be a comprehensive survey of the literature. Instead, we focus on illustrative examples where the sense of chiral induction has been established experimentally. We aim to provide a simple, general framework for understanding mechanisms of chiral induction that can be applied using intuitive ideas from conformational analysis. Where appropriate, we also use computational modeling of small structural subunits, but only simple torsional potential energy surfaces and geometry optimizations that do not require deep expertise.

While many of the original reports of these examples include models or rationalizations of the observed twist sense, in most cases we have reevaluated the results in the context of a general strategy, described below. The resulting mechanisms highlight the importance of considering short‐ and long‐range interactions to design foldamers with controlled helical structures.

## 2 General Mechanism of Chiral Induction

This review is organized around the simple idea that most mechanisms of chiral induction in foldamers can be described in two conceptual phases. First, we consider short‐range interactions between the controller group and the repeat unit to which it is directly attached. These interactions influence the orientations of freely rotating σ bonds and are often based on well‐understood conformational preferences, including standard functional group behavior (including dipole–dipole interactions, stereoelectronic effects, etc.) or deliberately engineered interactions such as hydrogen bonds. They ultimately establish the preferred orientation of the ligands about the controller group's chirality center(s) with respect to the backbone. These short‐range interactions are amenable to straightforward ab initio modeling, unlike full foldamer systems which are often too large and would require the consideration of too many conformers for a comprehensive analysis (i.e., for a system with *N* freely rotating σ bonds, there are typically $∼N^{3}$ conformers to consider). Second, we consider long‐range interactions that extend beyond the immediate neighboring units and describe the influence of the controller group on parts of the oligomer's backbone that have folded back into its proximity. These interactions will usually occur between the ligands and backbone repeat units one turn farther along the helix. Of course, in reality both the short‐ and long‐range interactions act simultaneously, but they are usually sufficiently independent to be considered separately.

This framework is simple, and perhaps obvious, and implicitly underlies most reported models of chiral induction. But it is helpful to consider it explicitly. In many of the examples below, the molecular design is primarily at the level of the short‐range interactions, and includes hydrogen bonding, steric effects, and bond torsional effects to guide the controller group. The long‐range interactions are generally simpler, often steric, although thorough understanding of both is required to determine the overall helicity.

Note that the order in which we consider these interactions, first short‐range and then long, does matter. We will usually orient the controller group and then consider the two possible helical twist senses, deriving (quasi‐)Newman projections of the favorable and unfavorable geometries. The predicted favorable conformer should capture the essential features of the actual favored conformer of the foldamer. As such, this approach allows the preferred twist sense to be predicted or at least rationalized. The unfavorable conformers shown below are not, however, necessarily the preferred geometry of that particular twist sense for the helix. This diastereomer is by definition strained, and that strain could manifest in reorientation of the controller group. This geometry is less likely to be known and will not be considered in detail. The emphasis here is on developing simple, intuitive models for the “best” conformers, as opposed to detailed‐ folding energy landscapes.

## 3 Controller Groups at the Termini

### 3.1 Aromatic Oligoamide Foldamers

Aromatic oligoamides are arguably the most prominent class of nonpeptidic foldamers.^[^ ^47^ ^]^ Among these, Huc's quinoline‐based foldamers stand out for their ability to form stable, well‐organized helical structures, primarily driven by a network of hydrogen bonds along their backbones.^[^ ^47^, ^48^, ^49^, ^50^ ^]^ These foldamers have been used in diverse functional systems, including as therapeutics,^[^ ^51^ ^]^ for molecular recognition,^[^ ^52^ ^]^ as molecular switches,^[^ ^18^ ^]^ and in molecular electronics.^[^ ^11^ ^]^ Huc's exploration of the control of helical handedness in aromatic oligoamides^[^ ^34^, ^53^ ^]^ provides significant contributions to understanding how noncovalent interactions, such as hydrogen‐bonding and steric effects, can be used to control foldamer twist sense.

**Figure 1:**
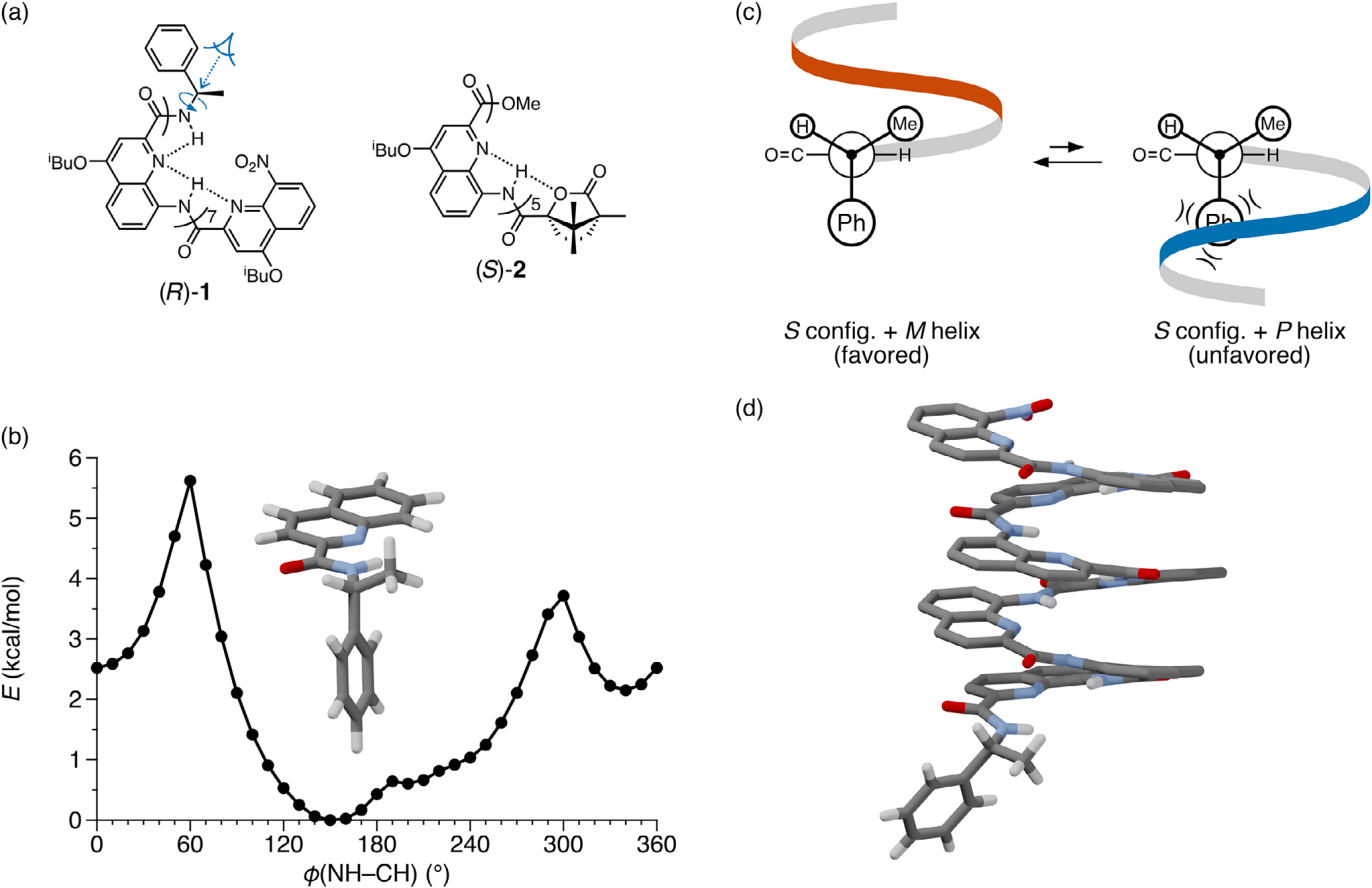
a) Huc's aromatic oligoamides (*R*)‐**1** ^[^ ^34^ ^]^ and (*S*)‐**2**.^[^ ^53^ ^]^ b) Potential energy surface and optimized geometry of the terminal subunit of (*R*)‐**1** for rotation about the C*─N bond (the dihedral angle is measured between the hydrogens; PCM(CHCl_3_)/ωB97X‐D/cc‐pVDZ). c) Newman projections viewing the C*─N bond of (*R*)‐**1**. A left‐handed (*M*) helix is directed away from the large phenyl group, whereas the right‐handed (*P*) helix would experience a steric clash. d) Crystal structure of (*R*)‐**1** (side‐chains and nonhydrogen‐bonding backbone H atoms removed for clarity).^[^ ^34^ ^].^

A representative example^[^ ^34^ ^]^ of an oligoamide foldamer is shown in Figure 1a. The twist sense of octamer (*R*)‐**1** is controlled by the 1‐phenylethylamine^[^ ^54^ ^]^ moiety attached at the oligomer's C‐terminus. Although (*R*)‐**1** is a large compound with many potentially rotatable σ bonds, if we assume a fully realized hydrogen bonding network it actually has only two conformational degrees of freedom: rotation about the key C*─N bond (where C* is the chirality center) and the twist sense of the helix.

The original publication^[^ ^34^ ^]^ describing (*R*)‐**1** provides a detailed analysis of twist‐sense induction based primarily on careful analysis of solid‐state structures. Here, we reevaluate this system by considering the potential energy surface for rotation about the C*─N bond of the quinoline amide substructure shown in Figure 1b (see also Supporting Information). The plot has essentially two minima. In the global minimum, the phenyl group is orthogonal to the NH─CO amide plane with the C*─H bond directed toward the amide carbonyl (i.e., anti to the N─H). This preference constitutes the short‐range interactions and sets the orientation of the ligands with respect to the point of attachment to the helix.

Long‐range steric interactions with the rest of the helix then predict the twist sense in this system. Newman projections viewing down the C*─N bond (see Figure 1a) are shown in Figure 1c. The hydrogen bonding network in **1** requires that, from the perspective of the viewer, the helix must proceed away from the amide carbonyl (to the right) and initially twist out of the page. A left‐handed *M* helix would then be directed upward, on the side of the chirality center with the small (H) and medium (Me) ligands, which are nearly in the plane of the first quinoline unit. A right‐handed *P* helix would be directed downward, directly into the large (Ph) ligand. Since this should lead to a steric clash, the prediction is that the long‐range interactions are minimized for the *M* helix in this system. This result is indeed what is observed experimentally for (*R*)‐**1** (82% d.e. in CDCl_3_).^[^ ^34^ ^]^ The predicted favored conformation is similar to what is found in the crystal structure of the racemate, shown in Figure 1d. The Huc group reported a variety of other substituents about the chirality center, and the model in Figure 1c is generally found to predict the correct twist sense.

Another oligoamide, foldamer (*S*)‐**2** (Figure 1a), emphasizes the importance of the short‐range interactions in determining twist sense.^[^ ^53^ ^]^ In this case, hydrogen bonding to the ethereal oxygen of the (1*S*)‐camphanyl group further restricts the free rotation about the C*─C(O) bond, simplifying the analysis as the twist sense is effectively the only conformational degree of freedom. A model analogous to that in Figure 1c predicts that the *P* helix should be preferred, as is observed. Notably, the d.e. (99%) is much higher than that for **1**, which has a freely rotating controller group. The mechanism of chiral induction for **2** has been studied in detail by molecular dynamics.^[^ ^55^ ^]^ The favored conformer indeed best accommodates the bulky dimethylmethylene group of the camphanyl group, minimizing unfavorable long‐range interactions.

**Figure 2:**
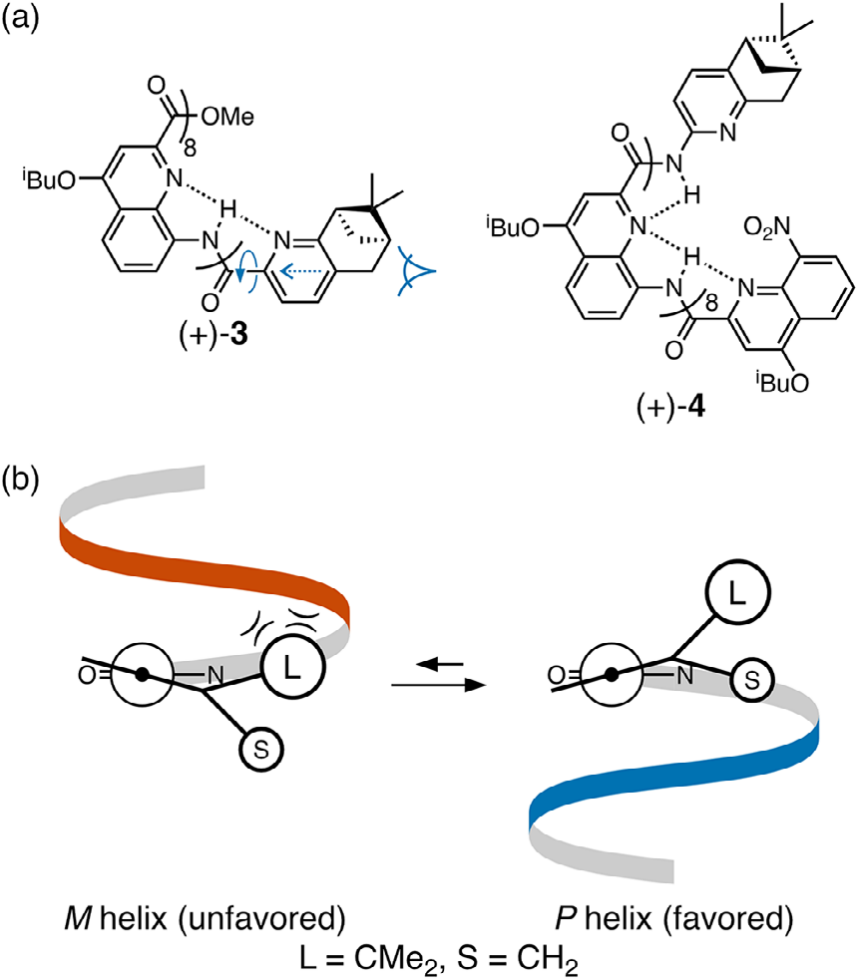
a) Jiang's aromatic oligoamides (+)‐**3** and (+)‐**4**.^[^ ^56^ ^]^ b) Newman projections for chiral induction in (+)‐**3**. The *P* helix is favored.

A related series of compounds developed by Jiang^[^ ^56^ ^]^ also highlights the importance of controlling short‐range interactions. Two representative examples, (+)‐**3** and (+)‐**4**, are shown in Figure 2a. These oligoamides incorporate the same chiral moiety, derived from β‐pinene, but attached at either the N‐ (**3**) or C‐termini (**4**). For **3**, the short‐range interactions are analogous to those in **2**: the controller group participates in the hydrogen bonding network responsible for folding, which sets its orientation with respect to the helix. Newman projections describing this system are shown in Figure 2b. The long‐range interactions are again steric: the *M* helix would lead to a clash with the larger dimethylmethylene group on the top face of the pinene moiety, whereas the *P* helix can be accommodated by the smaller methylene group, consistent with the experimental observation of quantitative formation of the *M* helix (as determined by NMR spectroscopy). The favored geometry is consistent with modeling results from Zhou,^[^ ^57^ ^]^ who has explored this system in detail.

The efficiency of chiral induction is dependent on the short‐range interactions in this system. For example, protonation of (+)‐**3**, which occurs preferentially at the pyridyl unit of the controller group, leads to a significant lowering of the d.e. as the key hydrogen bond is disrupted. Similarly, the sense of chiral induction is the same for C‐terminus‐substituted oligomer (+)‐**4** (*P* helix), but the efficiency is lower (73% d.e.). In this case, hydrogen bonding to the pyridyl nitrogen of the controller group is ineffective because of the restricted geometry (**4** would require a 4‐membered ring vs. 5‐membered for **3**). Oxidation of (+)‐**4** to the analogous *N*‐oxide, which should yield a more‐favorable geometry for hydrogen bonding, marginally increases the d.e. (80%).

Other oligoamides have been reported that make use of similar strategies for achieving chiral induction. For example, Jiang^[^ ^33^ ^]^ and Gan^[^ ^58^, ^59^ ^]^ have demonstrated chiral induction in several quinoline‐based foldamers by attaching chiral oxazolylaniline units at the C‐terminus. Similarly, Yang has reported chiral induction in herringbone helical foldamers.^[^ ^60^ ^]^

### 3.2 Aromatic Oligohydrazide Foldamers

**Figure 3:**
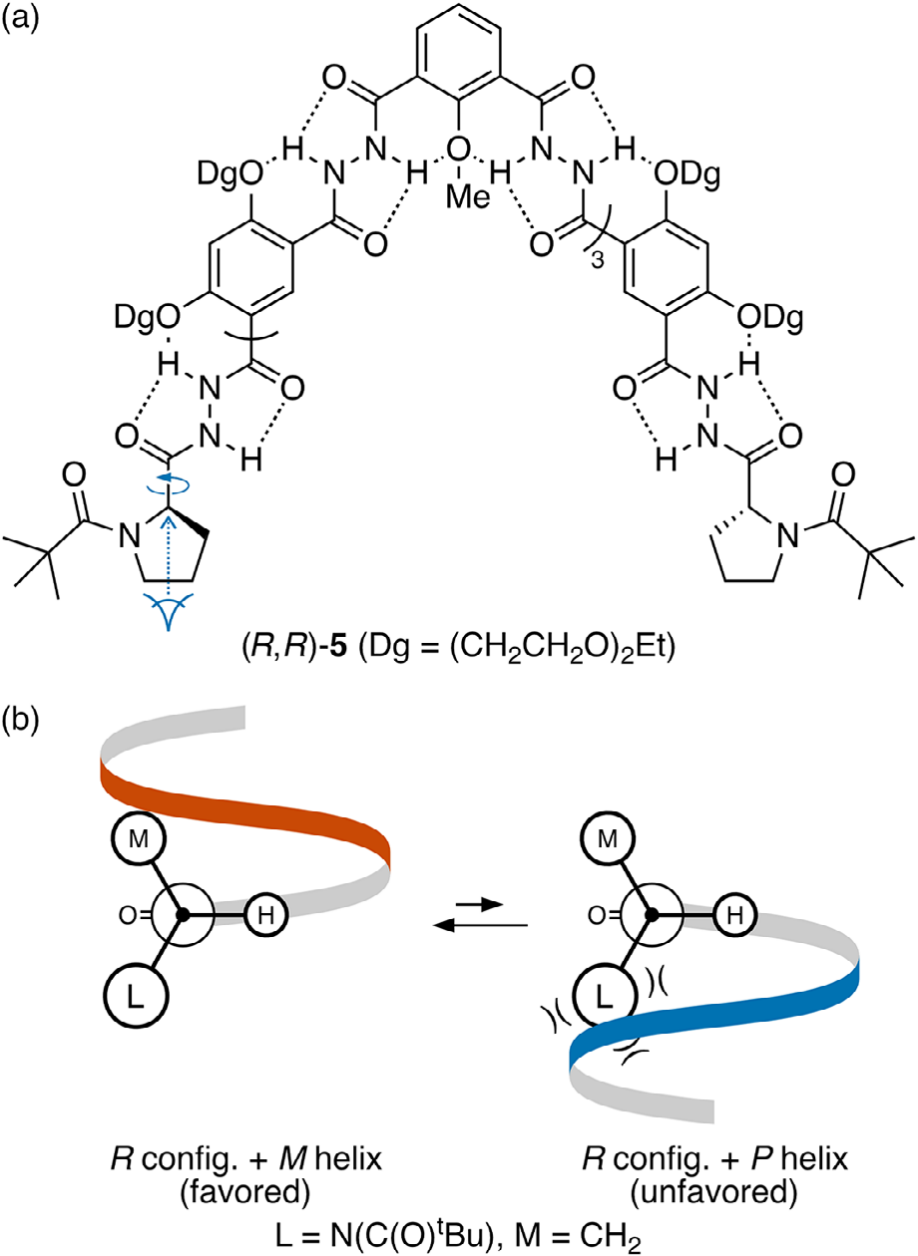
a) Li's oligohydrazide foldamer (*R*,*R*)‐**5**.^[^ ^61^ ^]^ b) Newman projections for chiral induction in (*R*,*R*)‐**5**. The *M* helix is preferred.

Li reported the first series of hydrogen‐bonding‐driven hydrazide foldamers, which function as hosts for saccharides.^[^ ^23^ ^]^ Oligomer (*R*,*R*)‐**5**, in Figure 3a, is a representative example.^[^ ^61^ ^]^ Here, chiral induction has been achieved by attaching chiral proline units at the termini of the twofold‐symmetric backbone. Like the aromatic oligoamides discussed above, these oligomers have, in principle, many rotatable σ bonds, but an extended hydrogen bonding network along the backbone directs their folding. Consequently, in (*R*,*R*)‐**5**, there are again only two important degrees of conformational freedom: the twist sense of the helix and rotation about the C*─C(O) bond to the chirality center (shown in Figure 3a).

A crystal structure reported in the original paper^[^ ^61^ ^]^ established that the hydrogen ligand attached to the chirality center prefers to be anti to the carbonyl. We have confirmed that this should also be true in solution by calculating a potential energy surface for rotation about the C*─C(O) bond in a small model system (see Supporting Information). This preference then is the short‐range interaction that establishes the orientation of the ligands about the chirality center with respect to the first repeat unit of the oligomer. Newman projections viewing down the key bond are shown in Figure 3b. As drawn, the hydrogen bonding network requires that the helix proceed to the right and then outward toward the viewer. An *M* helix, which progresses upward, is then predicted to be favored, as the alternative *P* helix requires a steric clash with the large (N(C(O)^t^Bu)) ligand. This model prediction aligns with results from molecular dynamics simulations,^[^ ^61^ ^]^ which suggested that the right‐handed *P* helix is energetically unfavorable by 12–15 kcal/mol compared to the left‐handed *M* conformation for *R* chirality centers.

Controlling the twist sense in (*R*,*R*)‐**5** has a strong effect on its binding to (chiral) guests, emphasizing an important application of twist sense control. Oligomer (*R*,*R*)‐**5** binds to ʟ‐glucose with a binding constant (12000 M^−1^) 40‐fold higher than that to ᴅ‐glucose (300 M^−1^). In the best case, a shorter foldamer, the binding constants differ by a factor of 144.

### 3.3 Oligoindole Foldamers

**Figure 4:**
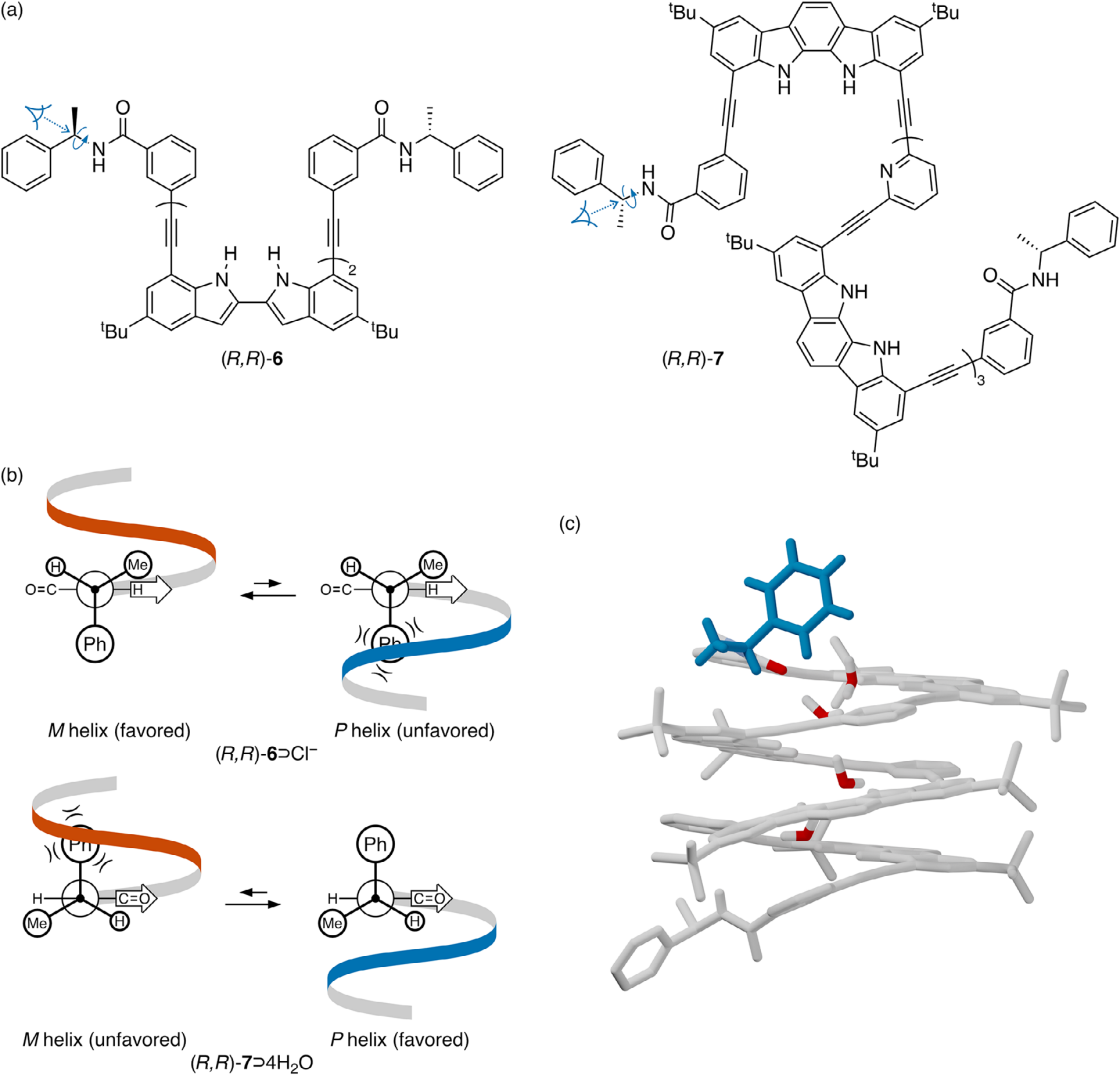
a) Structures of Jeong's (*R*,*R*)‐**6** ^[^ ^64^ ^]^ and (*R*,*R*)‐**7**.^[^ ^65^ ^]^ b) Chloride‐assisted folding of (*R*,*R*)‐**6** leads to the left‐handed helix, whereas water‐assisted folding of (*R*,*R*)‐**7** gives the right‐handed helix despite the use of the same (*R*)‐1‐phenylethylamine moiety to control the twist sense. The arrows indicate the preferred orientations of ligands because of hydrogen bonding to the guests. c) Crystal structure of (*R*,*R*)‐**7**.^[^ ^65^ ^]^ Note the controller group at the top in blue, which conforms to the Newman projection. Its carbonyl O atom and those in the water guests are shown in red. The controller group at the bottom is involved in an intermolecular hydrogen bond to another foldamer molecule and so its orientation does not reflect solution‐phase behavior.

Jeong has reported oligoindole foldamers, such as (*R*,*R*)‐**6**, shown in Figure 4a, that fold into helical conformations only after binding to anions in nonpolar solvents.^[^ ^62^, ^63^ ^]^ Helical bias was induced in these foldamers by attaching chiral units at the termini, opening up potential applications in chiral recognition and helicity‐switching systems.^[^ ^64^ ^]^ For (*R*,*R*)‐**6** ⊃ Cl^−^, binding to chloride drives the formation of the helix by directing all N─H bonds inward.^[^ ^64^ ^]^ The chloride also establishes the short‐range interactions in this system; the amide N─H bond to the controller group is similarly directed inward, which leaves only rotation about the C*─N bond to establish the spatial orientation of the chirality center's ligands. The behavior of this bond is similar to that in Huc's system (Figure 1); the small hydrogen prefers to be syn to the amide carbonyl, with the phenyl group approximately orthogonal to the plane of the amide. From there, the Newman projections in Figure 4b predict that the left‐handed *M* helix should be favored on the basis of sterics, consistent with ab initio calculations in the original report (which predict the *P* helix for the (*S*,*S*) enantiomer)^[^ ^64^ ^]^ and with our own TD‐DFT simulations of the experimental CD spectra using a simplified model oligomer (see Supporting Information). This system only gives significant CD signals assigned to backbone absorbances in the presence of chloride, underlining the role of hydrogen bonding in driving the folding and chiral induction.

Later work from Jeong shows that the short‐range interactions can be used to control the twist sense. A series of indolocarbazole–pyridine hybrid foldamers, such as (*R*,*R*)‐**7**, also adopt a helical conformation with a tubular cavity.^[^ ^65^, ^66^, ^67^ ^]^ Although the backbones of (*R*,*R*)‐**6** and (*R*,*R*)‐**7** are different, the mechanisms of chiral induction in the foldamers should be comparable. Similar to the binding of chloride to (*R*,*R*)‐**6**, water molecules can be bound within the cavity of (*R*,*R*)‐**7** by forming multiple hydrogen bonds.^[^ ^65^ ^]^ However, foldamer (*R*,*R*)‐**7** ⊃ 4H_2_O differs significantly from (*R*,*R*)‐**6** ⊃ Cl^−^ in the nature of the short‐range interactions directing the controller groups. Unlike (*R*,*R*)‐**6** ⊃ Cl^−^, in (*R*,*R*)‐**7** ⊃ 4H_2_O the guest water molecules act as hydrogen bond *donors* to the controller groups, causing the carbonyl group of the amide to face toward the helix instead of the N─H. As shown in Figure 4b, the reversal of the hydrogen bonding to the amide inverts the predicted twist sense of the oligomers, with the *P* helix now expected for the *R* controller group. Indeed, this has been confirmed by crystallography,^[^ ^65^ ^]^ shown in Figure 4c, which shows that the most‐stable conformation about the C*─N bond is as expected, with the phenyl group directed away from the helix. The d.e. for (*R*,*R*)‐**7** ⊃ 4H_2_O is 70% in CD_2_Cl_2_. Replacing the phenyl groups with larger naphthyl groups increases the d.e. to 92% in CD_2_Cl_2_ (96% in (CD_2_Cl)_2_), suggesting that the primary driver of chiral induction is steric.

### 3.4 Oligo(*ortho*‐Phenylene) Foldamers

We have explored *ortho*‐phenylenes as an unusual class of aromatic foldamers where the folding is driven primarily by arene–arene stacking interactions between the aromatic rings.^[^ ^68^ ^]^ In these systems, unlike the previous examples, there are not directional (e.g., hydrogen bonding) interactions that direct the orientation of each nominally freely rotatable σ bond. Rather, folded helical conformers are favored because they maximize the number of stabilizing arene–arene stacking interactions parallel to the helical axis. As a consequence, analysis of the short‐range interactions relevant to chiral induction is more complicated.

**Figure 5:**
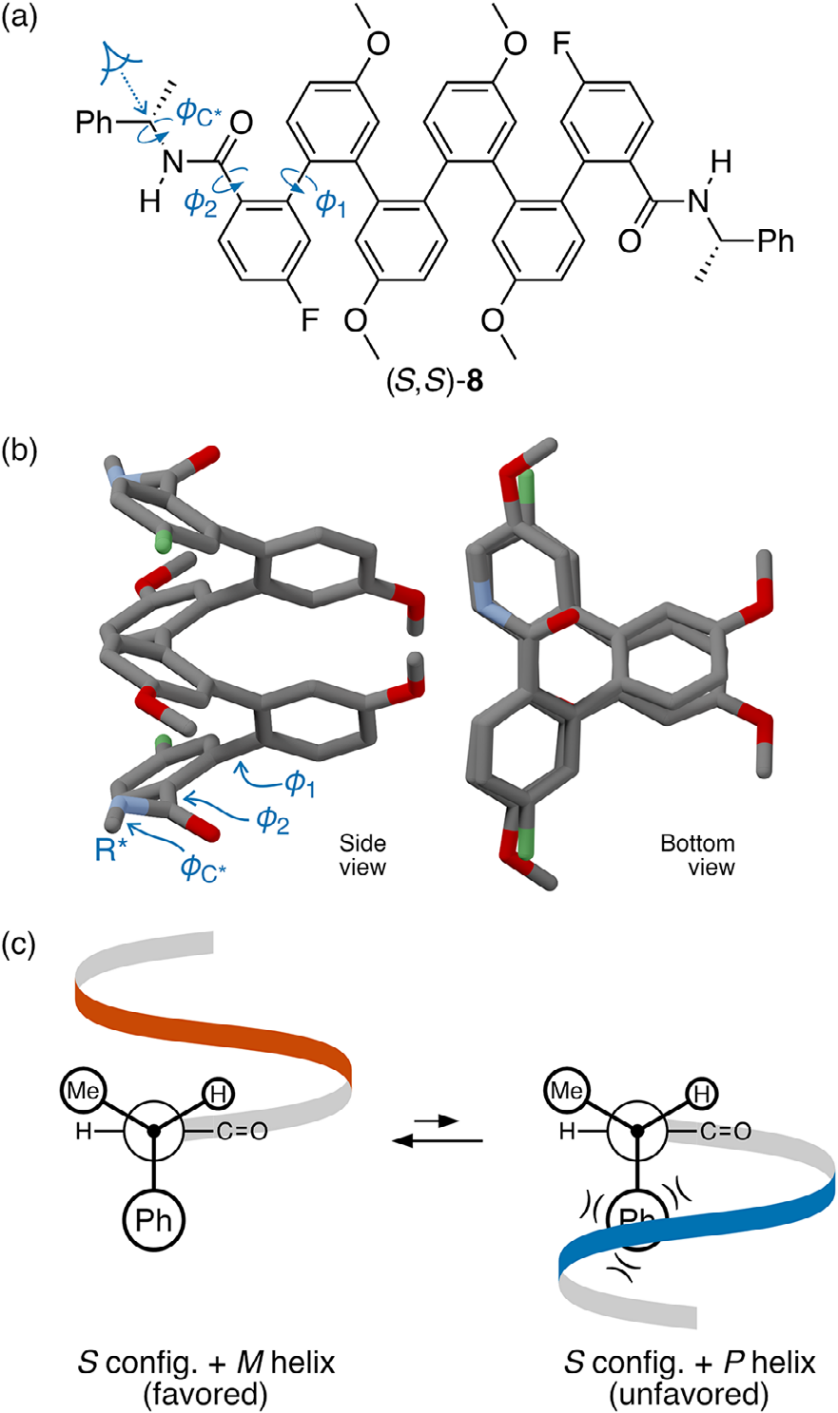
a) Oligo(*o*‐phenylene) (*S*,*S*)‐**8**, highlighting key bond torsions $\phi_{1}$, $\phi_{2}$, and $\phi_{C*}$.^[^ ^31^ ^]^ b) Optimized geometry of a simplified model of (*S*,*S*)‐**8** in its favored conformation (controller group replaced by methyl). c) Newman projections predicting that the *S* configuration of the chiral moiety should favor the *M* helix.

*o*‐Phenylene hexamer (*S*,*S*)‐**8** in Figure 5a provides a representative example.^[^ ^31^ ^]^ This oligomer folds into a helix with three repeat units per turn (Figure 5b). Three key bond torsions, $\phi_{1}$, $\phi_{2}$, and $\phi_{C*}$, do not affect the overall folding state of the *o*‐phenylene backbone, but are key to understanding how the controller group is oriented. Unlike the previous examples, there are no hydrogen bonds to the amide group, but it is assumed to also be trans because of well‐known preferences (i.e., the two substituents are on opposite sides of the C(O)─N(H) bond).^[^ ^69^, ^70^ ^]^ Torsion $\phi_{1}$ determines the overall orientation of the amide with respect to the helix, either “in” or “out”. In the “in” orientation the group follows the path of the helix and lies beneath the third repeat unit along the backbone (as in Figure 5b). Torsion $\phi_{2}$ determines the orientation of the N atom (or carbonyl) of the amide group, which can be either “N‐in” (pointed toward the helix interior) or “N‐out” (pointed toward the helix exterior, as in Figure 5b). Finally, $\phi_{C*}$ follows the analysis of similar systems above (i.e., phenyl ligand orthogonal to plane of amide, H syn to carbonyl, as in Figure 1b).

In principle, determining the favored values of $\phi_{1}$ and $\phi_{2}$ is a manageable problem for ab initio predictions. In practice, a combination of NMR spectroscopy and computational results determined that the most likely favored conformation was the in/N‐out configuration shown in Figure 5b.^[^ ^31^ ^]^ A purely theoretical approach could have been used, as DFT predictions are nearly in agreement. This was the second‐most stable conformer according to calculations at the PCM(CHCl_3_)/ωB97X‐D/cc‐pVDZ level for a simplified model.^[^ ^31^ ^]^

With the short‐range interactions established, using long‐range interactions to predict the preferred twist sense is straightforward and follows the above examples, as illustrated in Figure 5c. Steric interactions suggest that the *S* controller group should favor the *M* helix. This is indeed observed experimentally for (*S*,*S*)‐**8**, where CD spectroscopy shows that the *M* helix predominates for this system. The in–in/N‐out conformer gives 82% d.e. of the dominant twist sense. Consistent with steric effects determining the outcome, the d.e. is somewhat higher if the large group is ^t^Bu instead of Ph (>90%), lower if it is the smaller ^i^Pr (16%), and comparable for cyclohexyl (60%).

**Figure 6:**
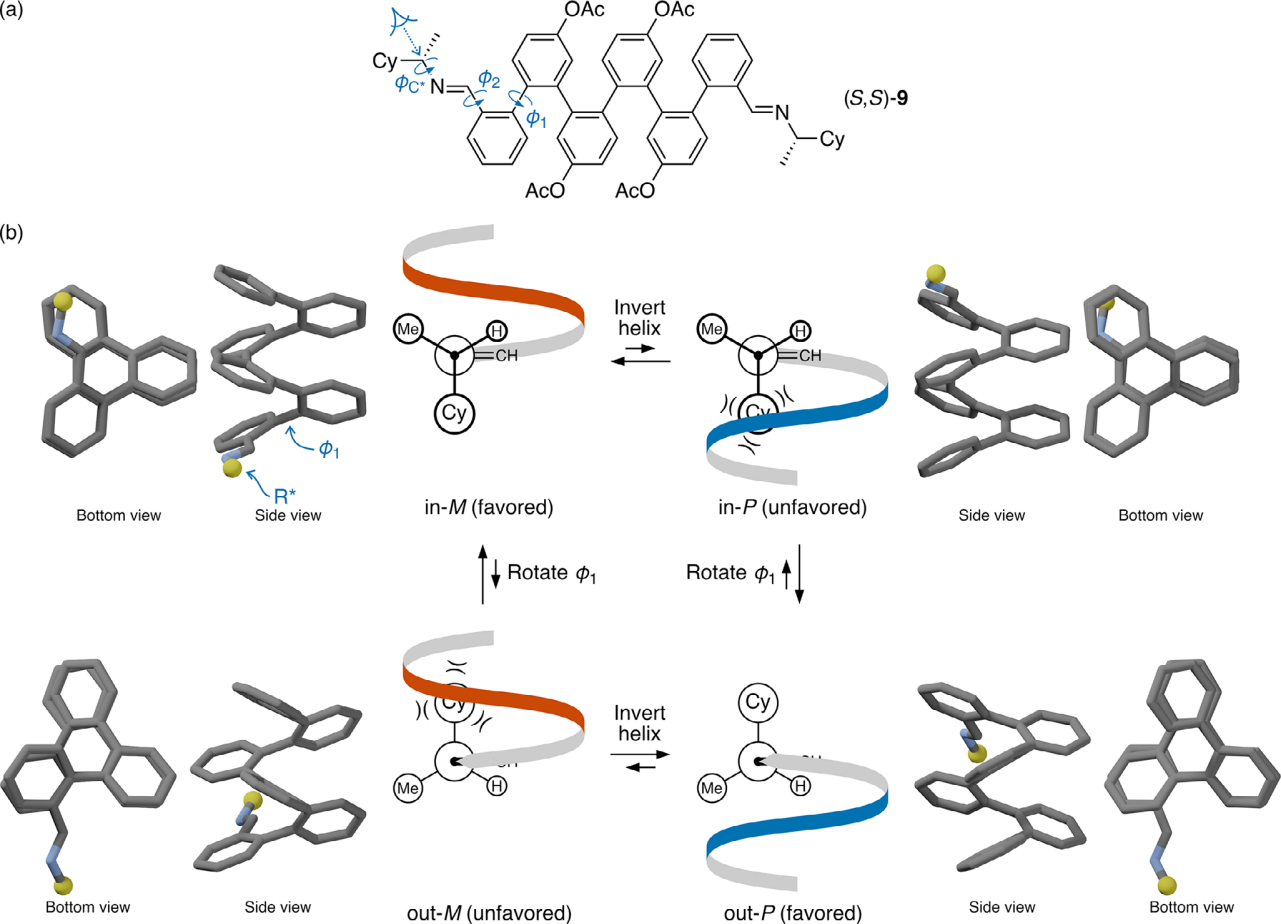
a) Oligo(*o*‐phenylene) (*S*,*S*)‐**9** (Cy = cyclohexyl).^[^ ^30^ ^]^ b) Newman projections showing that rotation about $\phi_{1}$ in this system is expected to yield inversion of the helix for the same configuration of the chirality center. In the geometries, the controller group is simplified to a yellow sphere.

In related work, our group has examined *o*‐phenylenes with chiral imine groups at the termini, such as (*S*,*S*)‐**9** in Figure 6a.^[^ ^30^ ^]^ The analysis of these systems is similar to that of amide (*S*,*S*)‐**8** (Figure 5), but with an important difference. With the imines, $\phi_{1}$ is not well controlled, with both in or out orientations observed simultaneously by NMR spectroscopy. Careful analysis of NMR and CD spectra allowed the populations of the different conformers to be determined and the twist senses assigned for each. For example, for (*S*,*S*)‐**9**, the conformer populations are 24% in–in, 59% out–out, and 17% in–out. The in–in conformer gives a predominant *M* twist sense for the helix (68% d.e.), whereas, curiously, the out–out conformer favors the opposite *P* twist sense (54% d.e.). For the in–out conformer, the *P* twist sense predominates, but only barely (8% d.e.)^[^ ^71^ ^]^

Figure 6b shows that this behavior can be explained by analyzing the short‐ and long‐range interactions of the controller group. DFT calculations suggest that the torsion $\phi_{2}$ behaves similarly to that in the amides; the N atom of the imine prefers to be directed N‐out, away from the helix. Key torsion $\phi_{C*}$ is also similar; the large ligand, in this case a cyclohexyl, is orthogonal to the plane of the imine (C─N═C) with the small hydrogen syn to the imine carbon. Analysis of the $\phi_{1}$ “in” conformers, shown at the top of Figure 6b, is directly analogous to the analysis of the amides in Figure 5b, predicting the *M* helix for the *S* configuration of the controller group on the basis of long‐range steric interactions. Rotation about $\phi_{1}$ to the “out” conformers, at the bottom of Figure 6b, changes the orientation of the controller group with respect to the helix. Now the steric clash occurs for the *M* helix and the *P* twist sense is predicted to be favored, as is observed. Not surprisingly, chiral induction tends to be less efficient when the controller group is directed outward, where it arguably behaves more like a side‐chain (see below).

Like Jeong's work in Figure 4, this system demonstrates that chiral controller groups can be “ambidextrous”, yielding opposite handedness depending on their orientation with respect to the helix. Interestingly, in many cases the d.e. in the *o*‐phenylenes’ unsymmetrical in/out conformers was found to be very low and near the average of the d.e.’s for the two symmetrical conformers, indicating that the controller groups at either end of the foldamer exert their influence largely independently. As would be expected, the degree of chiral induction depends on the steric demand of the large ligands, at least when directed inward (e.g., ^t^Bu > ^i^Pr > Et). A similar system, in which the twist sense of a phenylene ethynylene foldamer is inverted on binding to an achiral guest, has been reported by Katoono.^[^ ^72^ ^]^

### 3.5 Oligooxymethylene Foldamers

**Figure 7:**
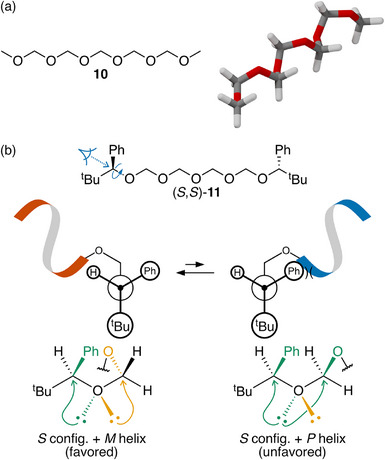
a) Helical folding of methyl‐terminated oligooxymethylene **10**. The geometry (*P* helix) is viewed down the methyl─O bond to match the Newman projection below. b) Newman projections for chiral induction in Noe's oligooxymethylene (*S*,*S*)‐**11** and anomeric interactions involving the oxygen atom connected to the chirality center.^[^ ^74^ ^]^ The *M* helix is preferred in this system.

Paraformaldehyde (i.e., polyoxymethylene) has long been known to adopt a helical conformation, shown in Figure 7a.^[^ ^73^ ^]^ Its folding results from stereoelectronic effects: synclinal (gauche) conformations are favored over antiperiplanar because of the anomeric effect, and torsions of the same sign along the backbone, leading to a helix, are preferred because they allow each of the oxygen atoms’ lone pairs to participate in one n−σ* interaction.^[^ ^74^ ^]^

Noe has explored the preference for helical handedness in these oligomers, attaching chiral alcohol moieties to their termini.^[^ ^74^ ^]^ A representative example, (*S*,*S*)‐**11**, is shown in Figure 7b. The key degree of freedom for the short‐range interactions is rotation about the C*─O bond, which is determined by a combination of stereoelectronic effects and sterics. An anomeric interaction between one of the oxygen atom's lone pairs and the σ∗ orbital of the C*─Ph bond requires that the phenyl group be synclinal with respect to the oxymethylene chain.^[^ ^75^ ^]^ The *tert*‐butyl group must then be antiperiplanar with respect to the chain to minimize steric interactions. A potential energy surface generated for a simple model structure confirms this preference (see Supporting Information).

The Newman projections in Figure 7b show that the preferred twist sense can then be predicted. Viewed down the C*─O bond, a right‐handed *P* helix proceeds to the right, whereas the left‐handed *M* helix proceeds to the left (note also the geometry in Figure 7a, which shows the *P* helix drawn from the same perspective). The *P* helix should be disfavored by a steric clash with the phenyl group. There is also a notable short‐range interaction. The favored *M* helix allows both of the lone pairs on the oxygen atom attached to the chirality center to participate in anomeric interactions, as shown at the bottom of Figure 7b: one interacts with the σ∗ orbital of the C*─Ph bond, the other with that the neighboring C─O bond. For the unfavored *P* helix, one lone pair is uninvolved with the anomeric interactions, which is less favorable.^[^ ^75^ ^]^ This prediction of an *M* helix aligns with the X‐ray crystallography analysis of (*S*,*S*)‐**11**, which confirms the left‐handed helix for the (*S*)‐chiral center.^[^ ^74^ ^]^

### 3.6 Oligo(Aminoisobutyric Acid) Foldamers

**Figure 8:**
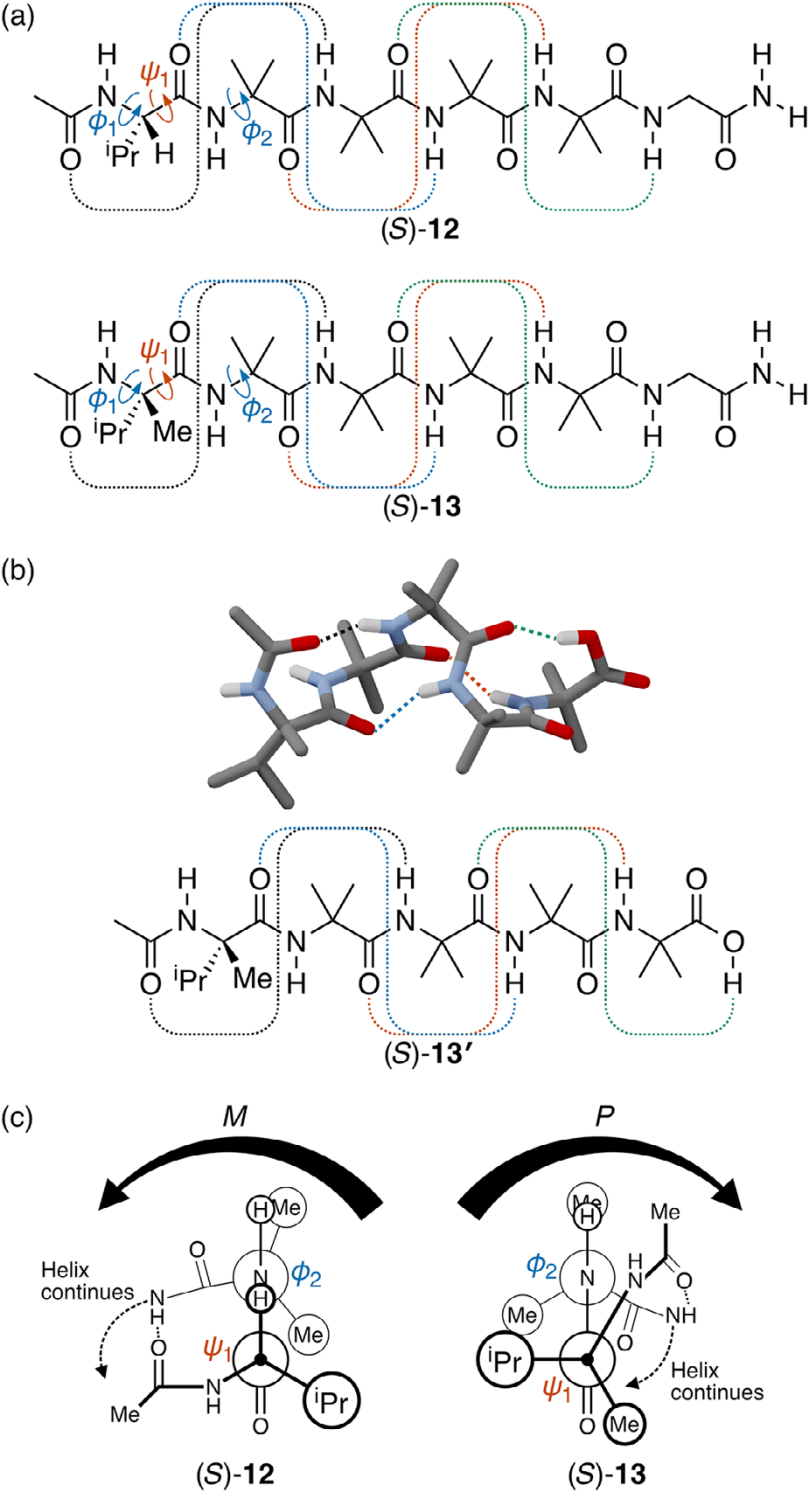
a) Clayden's Aib foldamers (*S*)‐**12** and (*S*)‐**13**, showing the key hydrogen bonds for folding.^[^ ^29^ ^]^ b) Crystal structure of (*S*)‐**13′**, showing the hydrogen bonding network.^[^ ^29^ ^]^ c) Newman projections of (*S*)‐**12** and (*S*)‐**13**. In both cases short‐range interactions establish the helicity of each oligomer at the N terminus, leading to *M* helicity for (*S*)‐**12** but *P* helicity for (*S*)‐**13**.

Inai made seminal contributions in demonstrating the induction of a preferred twist sense in peptides comprising only achiral amino acids.^[^ ^76^, ^77^, ^78^ ^]^ Direct chiral induction in the reported systems, however, appears to be weak: fivefold amplifications of the CD signals were possible by adding chiral additives that compete with the controller groups, suggesting that d.e.’s of the native foldamers are on the order of 20%.^[^ ^78^ ^]^ We therefore focus here on more‐recent work on (achiral) α‐aminoisobutyric acid (Aib) foldamers by Clayden. These peptides fold into 3_10_ helices stabilized by hydrogen bonds between every third repeat unit,^[^ ^79^ ^]^ as shown in Figure 8a. The attachment of chiral amino acids at the termini can then induce a favored twist sense to the oligomer, as in (*S*)‐**12**, incorporating the tertiary amino acid ʟ‐valine, and (*S*)‐**13**, incorporating the quaternary amino acid ʟ‐α‐methylvaline.^[^ ^29^, ^80^ ^]^ The hydrogen‐bonding pattern is shown in the solid‐state structure of oligomer (*S*)‐**13′**, which is closely related to (*S*)‐**13**, in Figure 8b. Despite their structural similarity, differing in only an H‐to‐methyl substitution, these two foldamers adopt opposite helical twist senses, *M* for (*S*)‐**12** and *P* for (*S*)‐**13**. These examples demonstrate that the long‐range interactions involving the controller groups can be attractive rather than steric.

The short‐range interactions in this system dictate the key dihedrals $\psi_{1}$ and $\phi_{2}$ (Figure 8a). Extensive crystallographic and NMR analysis^[^ ^29^ ^]^ has shown that the preferred conformation of (*S*)‐**12** is as shown in Figure 8c (left): here, $\psi_{1}$ ≈ +120° and $\phi_{2}$ ≈ +80° (and $\phi_{1}$ ≈ −60°), yielding a “type II” *β* turn. In this case, the large isopropyl ligand is sterically unencumbered, leaving only the small hydrogen ligand eclipsed with the rest of the chain ($\psi_{1}$). The long‐range interaction is then the hydrogen bond between the valine moiety's acetyl group and the N─H of the second Aib unit farther down the chain. This establishes *M* helicity that is then propagated along the backbone, because the next amide group must hydrogen bond to the downward‐facing carbonyl group as depicted in the figure.

In contrast, the quaternary amino acid in (*S*)‐**13** would introduce unfavorable steric interactions between the methyl group and the amide nitrogen were it to adopt the same type II β‐turn conformation. It instead favors the formation of a “type III” *β*‐turn as in Figure 8c (right). Here, the large isopropyl ligand is perpendicular to the plane of the amide, with the methyl group gauche to the amide carbonyl. By similar logic to (*S*)‐**12**, the long‐range hydrogen bond in (*S*)‐**13** then establishes *P* twist sense which is propagated along the oligomer. The efficiency of chiral induction is similar for both (*S*)‐**12** and (*S*)‐**13**, with d.e.’s on the order of 50%.^[^ ^29^ ^]^ Considerable further optimization of this system has been reported.^[^ ^81^ ^]^

**Figure 9:**
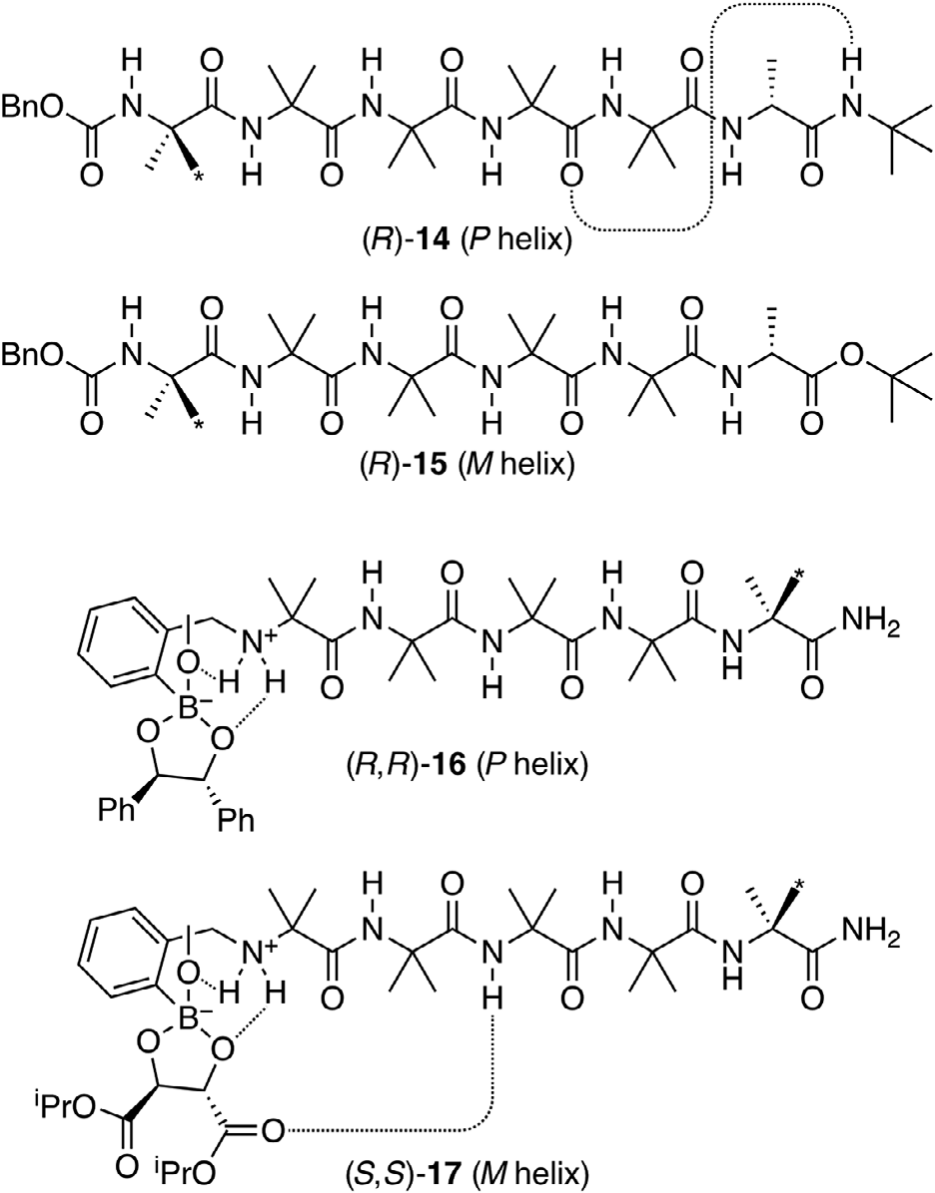
Aib foldamers demonstrating the importance of control over long‐range interactions (* indicates ^13^C labeling for NMR studies).^[^ ^82^, ^83^ ^].^

Foldamers **14**–**17** from the Clayden group, shown in Figure 9, further illustrate how long‐range interactions can control these systems. Their mechanisms of chiral induction are similar to those for (*S*)‐**12** and (*S*)‐**13** and will not be discussed in detail, although they are thoroughly described in the original works. Foldamers (*R*)‐**14** and (*R*)‐**15** are similar to (*S*)‐**12** and (*S*)‐**13**, except that the controller groups are at the C‐terminus.^[^ ^82^ ^]^ These oligomers are distinguished by a single N‐to‐O substitution near the chirality center. (*R*)‐**14** retains a hydrogen bond that approximately serves the same function as those in Figure 8c, and which generates a *P* helix. In (*R*)‐**15**, this long‐range interaction is not possible. Instead, the conformational preferences are dictated by dipole repulsion (a “Schellman motif”) and the helix is inverted (*M*).

Building on these mechanisms, Clayden has further explored long‐range structural communication in Aib foldamers, demonstrating their ability to mimic biological receptors. Oligomers (*R*,*R*)‐**16** and (*S*,*S*)‐**17** in Figure 9 exhibit helicity switching, making use of reversible boronate esterification to transmit chiral information from a boron‐based ligand binding site.^[^ ^83^ ^]^ Diol controller groups can be reversibly bound to the N‐terminal binding site, with the twist sense relayed to a reporter group at the C‐terminus. Oligomer (*R*,*R*)‐**16** can be produced through reaction of (+)‐hydrobenzoin with (*S*,*S*)‐**17**, displacing (−)‐diisopropyl tartrate, which results in a twist sense inversion, even though both diol moieties have the same absolute configuration (in terms of sterics), and even though both should hydrogen bond similarly to the nearby ammonium group. This switch from *M* to *P* twist sense suggests that the tartrate and hydrobenzoin derivatives influence the helix through differing long‐range interactions. The likely origin is a hydrogen bond between a tartrate carbonyl group and one of the two N‐terminal amide hydrogens that are not already part of the foldamer's hydrogen bonding network. The hydrogen bond overcomes steric repulsion, stabilizes a left‐handed β‐turn, and induces *M*‐helicity in the complex. In (*R*,*R*)‐**16**, the bulky phenyl ligands create steric hindrance, forming a steric block that induces *P*‐helicity.

## 4 Controller Groups at the Side‐Chains

Introducing controller groups into the side‐chains of foldamer backbones offers an alternative approach to achieving chiral induction, though this method typically generates a smaller d.e. compared to substitution at the termini. As illustrated in the examples above, terminal controller groups can have a significant impact on helical bias because they can directly participate in the interactions stabilizing folding, and because the difference between the two twist senses can often be thought of as the entirety of the foldamer being directed toward or away from key ligands. In contrast, the sides of a folded helix tend to present only subtle structural differences depending on the handedness. Another issue is that, as shown below, prediction of the twist sense will typically require modeling of at least one turn of the helix so as to understand the locations of substituents with which the ligands can interact.

### 4.1 Oligoindole Foldamers

**Figure 10:**
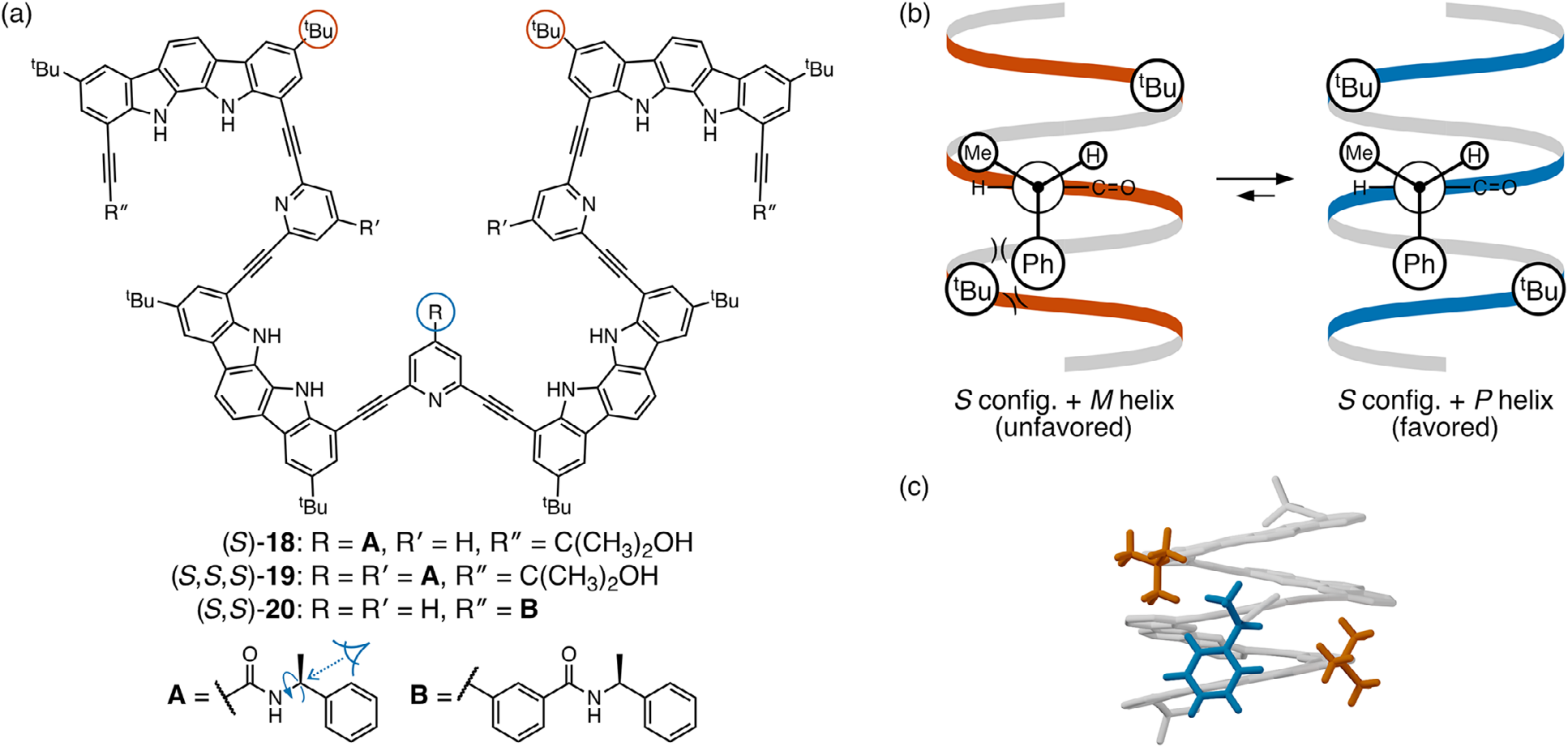
a) Jeong's oligo(indolocarbazole) foldamers **18**–**20**.^[^ ^36^ ^]^ b) Newman projection analysis of (*S*)‐**18**. c) Optimized^[^ ^36^ ^]^ structure of the favored conformation of (*S*)‐**18**. Hydrogens and tert‐butyl groups removed except for key groups. The controller group and key tert‐butyl groups are highlighted (these same groups are circled in (a)).

Jeong explored the relative abilities of controller groups to effect chiral induction from both the termini and side‐chains in indolocarbazole–pyridine hybrid foldamers **18**–**20**, shown in Figure 10a.^[^ ^36^ ^]^ The centrally located controller group in (*S*)‐**18** provides a useful representative example. Like (*R*,*R*)‐**7** (Figure 4), (*S*)‐**18** folds into a helix in nonpolar solvents driven by hydrogen bonding to bound water molecules. The side‐chain controller group cannot participate in these interactions. There are, however, still effectively only two degrees of freedom that must be considered: the twist sense of the helix and the rotation about the C*─N bond; the amide group should prefer to be coplanar with the pyridine ring to which it is attached, and otherwise rotation about the C─C(O) bond does not matter because of the twofold symmetry of the foldamer. As before, the small ligand (H) on the chirality center prefers to be syn to the amide carbonyl. Newman projections depicting the orientation of the ligands about the chirality center are shown in Figure 10b. Note that the structure of the amide places the chirality center slightly off‐center relative to the $C_{2}$ axis of the helix.

Establishing the long‐range interactions in systems like this can be tricky—the helix itself may not present many obvious interactions that would favor a particular twist sense. For (*S*)‐**18**, the *tert*‐butyl groups farther along the indolocarbazole backbone provide obvious bulk, but their positions relative to the controller group must be established through structural analysis of one turn of the foldamer helix. The cartoons in Figure 10b are based on MMFF optimizations reported in the original work;^[^ ^36^ ^]^ the favored conformation of (*S*)‐**18** is shown in Figure 10c. Folding brings two of the *tert*‐butyl groups close to the chirality center. As illustrated in Figure 10b, the *P* helix should be favored, as it places the large (Ph) ligand farther from the remote *tert*‐butyl groups, consistent with the original computational model. In the *M* helix, the Newman projection predicts a steric clash.

The *P* helix is indeed favored experimentally for (*S*)‐**18**; however, the difference in stability between the two twist senses is modest, with a 20% d.e. in toluene‐*d*_8_. The twist sense excess can be increased through the addition of more controller groups that act cooperatively, as in (*S*,*S*,*S*)‐**19**, which gives a 54% d.e. Chiral induction from the termini, as in (*S*,*S*)‐**20**, should follow a mechanism similar to that described in Figure 4c. This model predicts that the *S*,*S* isomer should favor the *M* helix, as is observed. While comparisons are difficult to make, chiral induction from the termini is somewhat more efficient, achieving a d.e. of 62% (keeping in mind that there are two controller groups in **20** compared to three in **19**).

### 4.2 Oligo(*ortho*‐Phenylene) Foldamers

**Figure 11:**
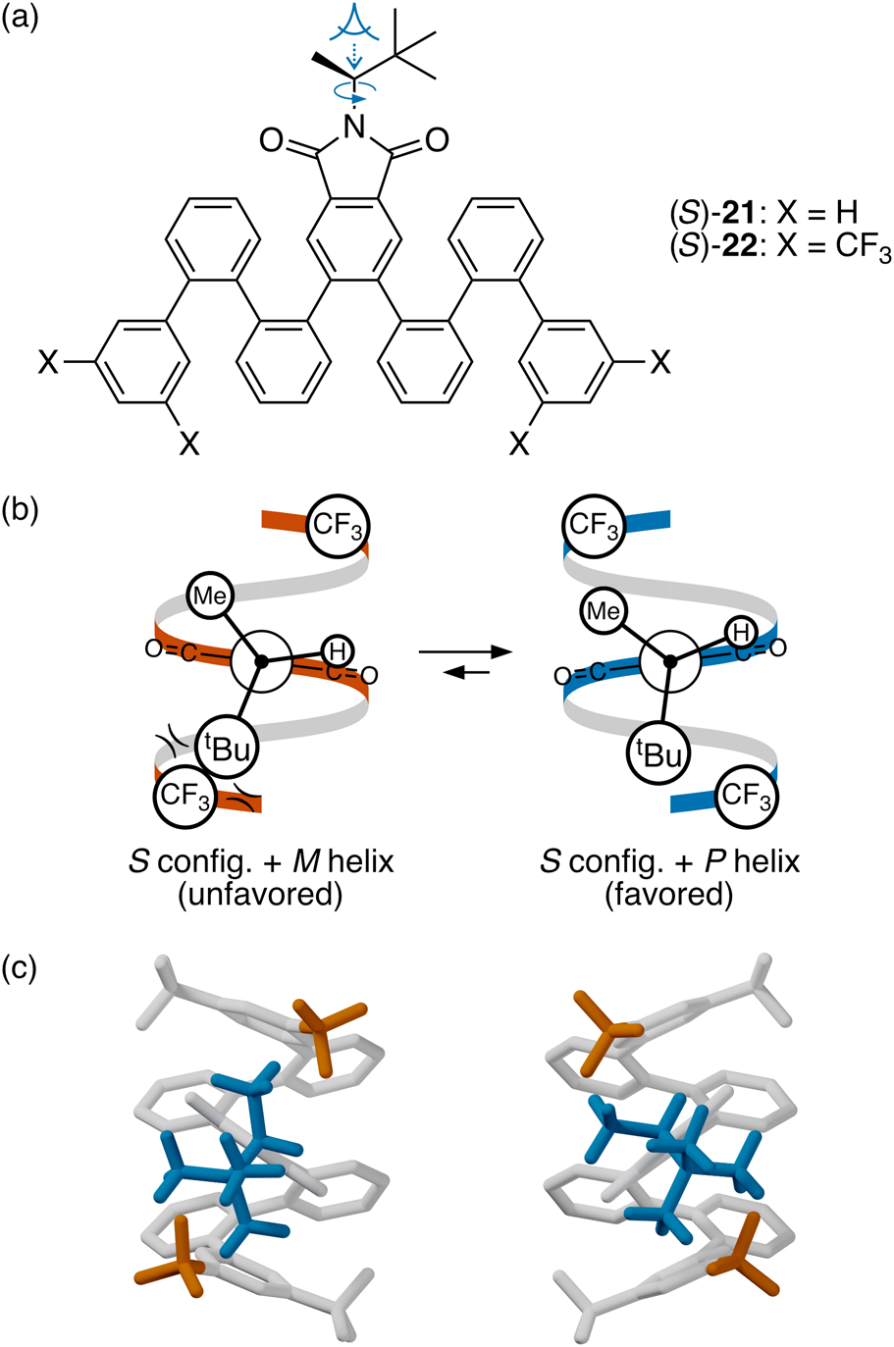
a) Oligo(*o*‐phenylenes) (*S*)‐**21** and (*S*)‐**22**.^[^ ^35^ ^]^ b) Newman projections illustrating chiral induction in (*S*)‐**22**. The *P* twist sense is favored. c) Optimized geometries^[^ ^35^ ^]^ of the two possible twist senses of (*S*)‐**22** (PCM(CHCl_3_/ωB97X‐D/cc‐pVDZ).

A recent example from our group highlights the importance of functionalizing the helix to better interact with the controller group's ligands.^[^ ^35^ ^]^ Oligomers (*S*)‐**21** and (*S*)‐**22**, in Figure 11, are both *o*‐phenylenes, with the same side‐chain controller group and differing only in substituents (X) at the termini. In (*S*)‐**21** (X = H), chiral induction is very weak, with a d.e. of only 4 ± 5%. Analysis of its CD spectrum suggests a small excess of the *M* helix. This poor result is unsurprising given that the chirality center is spaced out from the helix by the imide group, and that the helix itself presents only outward‐facing aromatic C─H bonds that would not be expected to interact strongly with the hydrogen, methyl, or *tert*‐butyl ligands. The helix's surface is, in essence, a featureless cylinder that provides little for the controller group to interact with.

In (*S*)‐**22**, the d.e. is much improved, to 69 ± 10% of the *P* helix. Although the trifluoromethyl groups are seemingly located far from the controller group, on folding they are placed right above it (two CF_3_ groups are included at each terminus to ensure that one is always facing the controller group). Again, there are only two degrees of conformational freedom for this structure: the twist sense of the helix and rotation about the C*─N bond. Similar to the amides, the small hydrogen ligand prefers to be syn to the carbonyl group of the imide; since the *o*‐phenylene backbone is twofold symmetric, it does not matter which one. The key long‐range interactions are then steric interactions between the large *tert*‐butyl ligand on the chirality center and the trifluoromethyl groups. Rationalizing the twist sense induction in this system again requires consideration of one helical turn of the foldamer in order to locate the CF_3_ groups relative to the controller group. The positioning shown in the Newman projections in Figure 11b is based on DFT models of the oligomers shown in Figure 11c. In the case of the *M* helix, the preferred orientation of the *tert*‐butyl ligand places it very close to the trifluoromethyl group, whereas for the *P* helix it is farther away. While not so easy to see in the models in Figure 11c, the difference is about 0.2 Å. The primarily steric nature of this interaction is supported by the behavior of other studied compounds: replacement of the CF_3_ groups with CH_3_ or the ^t^Bu ligand with ^i^Pr give smaller d.e.’s but consistent twist senses.

## 5 Summary and Outlook

Although this is not intended to be a comprehensive review, the examples presented above suggest a few general conclusions about chiral induction in helical foldamers. First, if the goal is the strongest twist‐sense induction, it is important to limit the number of freely rotatable bonds between the chirality center of the controller group and the helix. In many of the cases discussed above, to a first approximation (e.g., assuming that hydrogen bonding interactions are satisfied), there is only one degree of conformational freedom other than the helix's twist sense itself. This is especially important since in at least some cases the controller group's effect on the helix is strongly dependent on its exact positioning (e.g., Figures 4 and 5). Nevertheless, it is often not necessary to explicitly control the orientation of the controller group with designed interactions like hydrogen bonds; in many cases, the inherent preferences of freely rotatable groups are sufficient. Second, in most cases the long‐range interactions governing twist sense control are steric; there are likely opportunities to engineer attractive interactions that could help improve diastereoselectivity, particularly in cases of side‐chain controller groups where the helix may need to be functionalized to generate effective chiral induction. Third, terminal substitution tends to be more effective than side‐chain substitution in directing the twist sense. There is an important caveat, of course: a foldamer can have at most two termini for functionalization, whereas there may be many possible sites for side‐chain functionalization that can act cooperatively. Regardless, the design of the foldamer and its intended function will often dictate where the controller groups can be attached. For example, functionalization of foldamers at their termini may not be ideal for subsequent self‐assembly, as these groups can block key points of attachment.

What is accomplished by controlling the twist sense of foldamers? At a fundamental level, this is a necessary objective for the field. The goal of molecular folding is nanoscale structural precision, which requires that all aspects of the structure, including its handedness, be controlled. More pragmatically, folding provides a mechanism whereby the chiral information of inexpensive, readily available compounds can be amplified into larger‐scale structural asymmetry and translated into new function.^[^ ^84^ ^]^ These roles are exemplified by current applications in asymmetric molecular recognition^[^ ^6^, ^61^, ^85^ ^]^ or chiroptical materials.^[^ ^86^ ^]^ In these areas, folding allows structural complexity to be achieved with substantially less synthetic effort compared to alternatives, at least in principle. A folded oligomeric heterosequence can yield a more‐complex binding cavity compared to higher symmetry molecular cages,^[^ ^87^ ^]^ for example, and a folded conjugated backbone should be easier to synthesize than helicenes of similar lengths.^[^ ^88^ ^]^

We would argue that the dynamic nature of folding means that there are also unique opportunities in integrating chiral induction into dynamic properties and function.^[^ ^89^ ^]^ Foldamers can amplify smaller‐scale geometry changes from coupled molecular switches and motors.^[^ ^90^ ^]^ Abiotic foldamers remain mostly limited to secondary structure, and mostly limited to folding into helices. A sensible way to communicate information through a helix is via its twist sense.^[^ ^91^ ^]^ This strategy has been realized particularly effectively in Clayden's Aib foldamers. They have been used, for example, to transmit^[^ ^29^ ^]^ information through a phospholipid bilayer membrane via coupling of the twist sense to an azobenzene photoswitch.^[^ ^92^ ^]^ This area is as yet understudied with other foldamer backbones, and a detailed understanding of mechanisms by which controller groups are able to affect twist sense is critical to progress. For example, the ability of the same ambidextrous controller group to induce opposite twist senses depending on its orientation, as in Figures 4 and 6, offers opportunities to use switching of short‐range interactions to direct the overall foldamer structure.

## Supporting Information

Computational analysis of select systems.

## Conflict of Interest

The authors declare no conflict of interest.

## Supporting information



Supporting Information

## Data Availability

The data that support the findings of this study are openly available in Zenodo at https://doi.org/10.5281/zenodo.16415315, reference number 16415315.
