# Supplementary material for: Mechanisms of Chiral Induction to Foldamer Backbones
Source: Chemistry. 2025 Oct 8;31(61):e02433. doi: 10.1002/chem.202502433 (PMC12587027; doi:10.1002/chem.202502433)
Supplement: Supplementary file 1 — Supporting Information [file CHEM-31-e02433-s001.pdf]

Mechanisms of Chiral Induction to Foldamer Backbones

Supporting Information

*Govinda Prasad Devkota, Roshan Lama, and C. Scott Hartley**

Department of Chemistry & Biochemistry, Miami University, Oxford, Ohio 45056, USA

Table of Contents

Data availability **S2**

Computational analysis **S2**
Analysis of aromatic oligoamide (*R*)-**1** S2
Analysis of aromatic oligohydrazide (*R*,*R*)-**5** S2
Predicted CD spectrum of oligoindole (*R*,*R*)-**6** S4
Analysis of oxymethylene (*S*,*S*)-**11** S7

References **S8**

List of Figures

S1 Potential energy surface for rotation about the key bond in **S2**. S3
S2 Optimized geometry of **S2**. S3
S3 Optimized geometry of **S3**. S5
S4 Predicted CD spectrum of **S3**. S6
S5 Potential energy surface for rotation about the key bond in **S4**. S7

List of Charts

S1 Oligoamide model structure **S1**. S2
S2 Oligohydrazide model structure **S2**. S2
S3 Oligoindole model structure **S3**. S4
S4 Oxymethylene terminus model structure **S4**. S7

List of Tables

S1 Cartesian coordinates for the optimized geometry of **S1**. S2
S2 Cartesian coordinates for the optimized geometry of **S2**. S4
S3 Cartesian coordinates for the optimized geometry of **S3**. S6

Data availability

An archive of the raw data associated with this work is available through Zenodo at https://doi.org/10.5281/zenodo.16415315.

Computational analysis

All calculations were performed using Gaussian 16 rev. B.01.[1]

*Analysis of aromatic oligoamide (R)-**1***

The potential energy surface was generated for model structure **S1** (Chart S1) at the PCM($CHCl_3$)/ωB97-XD/cc-pVDZ level. The scans were started at $\varphi = 0°$ (measured between the two hydrogen atoms) and run in both directions in 10° increments, taking the lower energy at each point for the plot in Figure 1b.

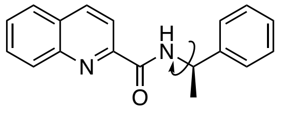

S1

Chart S1. Oligoamide model structure **S1**.

The minimum-energy geometry was then optimized without constraints, also at the PCM($CHCl_3$)/ωB97-XD/cc-pVDZ level. The final energy was −880.095944 E_h with no imaginary vibrational frequencies. The Cartesian coordinates of the optimized geometry are in Table S1.

```
C,0,-1.1313650714,-1.0184262646,0.0068035761
C,0,-1.9041489392,-1.9689290488,-0.7030268013
C,0,-3.2181356974,-1.6785695168,-0.9609561482
C,0,-3.760170879,-0.4452620838,-0.5149709107
C,0,-2.89689534,0.4416056801,0.1884842598
N,0,-1.5925618244,0.1364295267,0.4367874442
H,0,-3.853541317,-2.3808775329,-1.5047806418
H,0,-1.4314660623,-2.8968083882,-1.0208813441
C,0,-5.1104841164,-0.0651350749,-0.7397982939
C,0,-5.576135996,1.1435447475,-0.2836744533
H,0,-6.6140850164,1.431018189,-0.4585703018
C,0,-4.7146914496,2.0265470897,0.4168264766
H,0,-5.100587775,2.983283492,0.7726051471
C,0,-3.4049049722,1.6861122061,0.6487889219
H,0,-2.725911768,2.3503724034,1.1852268718
H,0,-5.7687911268,-0.7489896801,-1.2795942489
C,0,0.3227281828,-1.3378523165,0.2858569421
O,0,0.8355923185,-2.377093346,-0.1129035102
N,0,0.9638257986,-0.3906318795,1.0002926898
H,0,0.4136680508,0.4263232874,1.2481218226
C,0,2.3745859284,-0.4617305533,1.3212113516
H,0,2.6178533213,-1.5305271932,1.416288146
C,0,2.6256368821,0.2367040251,2.6585676395
H,0,2.0209915951,-0.2234409742,3.4536246208
H,0,3.6866240572,0.1642188399,2.9348936621
H,0,2.3679377817,1.3058074143,2.5899819224
C,0,3.2607220951,0.1185879507,0.2245664776
C,0,2.7907428537,1.080648011,-0.6719580023
C,0,4.5967212194,-0.2870911047,0.1380838711
C,0,3.6400654493,1.6306851079,-1.6328239046
H,0,1.7462150401,1.3954345501,-0.6307056035
C,0,5.4476502062,0.2601820655,-0.8197008426
H,0,4.9743074333,-1.0473268357,0.8272062029
C,0,4.9711393919,1.2242783128,-1.7092141268
H,0,3.2559237426,2.3793385142,-2.3285589504
H,0,6.485988596,-0.0722755737,-0.8767247469
H,0,5.6344844069,1.6520279535,-2.4631752147
```

Table S1. Cartesian coordinates for the optimized geometry of **S1**.

*Analysis of aromatic oligohydrazide (R,R)-**5***

Similar to the analysis of **S1**, a potential energy surface was generated for rotation about the key bond in **S2** (Chart S2), running the calculation forward and backward at the ωB97-XD/cc-pVDZ level in 10° increments. The PES is shown in Figure S1.

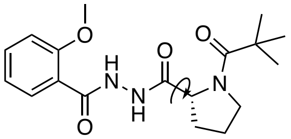

Chart S2. Oligohydrazide model structure **S2**.

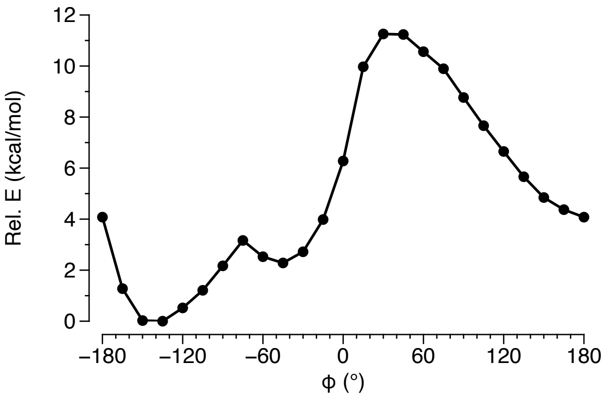

Figure S1. Potential energy surface for rotation about the key bond in **S2**.

The minimum-energy geometry was then optimized without constraints at the ωB97-XD/cc-pVDZ level. The final energy was −1165.86687722 E_h and there were no imaginary vibrational frequencies. A model of the optimized geometry is in Figure S2 and its Cartesian coordinates of the optimized geometry are in Table S2. The model is consistent with the proposed hydrogen bonding network in (*R*,*R*)-**5** and shows that the small H ligand should be oriented anti to the carbonyl group in the favored geometry.

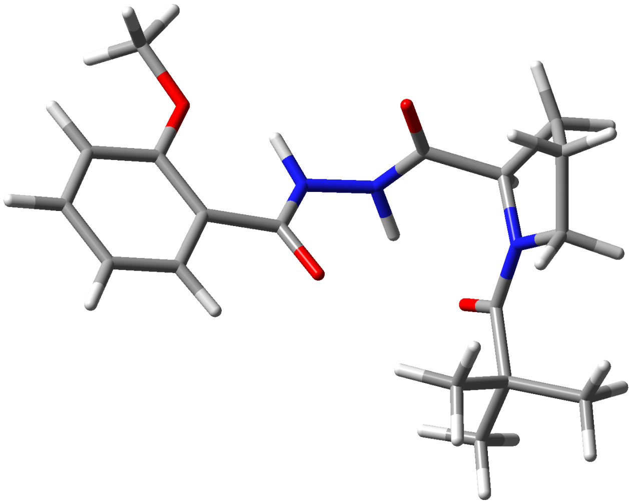

Figure S2. Optimized geometry of **S2**.

C,0,-2.1765254622,-1.9081688575,-0.3232378749	C,0,-4.8815852727,1.6741452923,0.2238465237
C,0,-2.7402377118,-2.9612677246,0.6258363831	H,0,-4.9715289558,1.219545526,1.2182588442
C,0,-2.9221416817,-2.1805853682,1.9275926131	H,0,-5.3050912721,2.687767179,0.2965344399
C,0,-3.4407132436,-0.8281126297,1.4447520505	H,0,-5.5083130247,1.1026541902,-0.4791865605
H,0,-2.4438457712,-2.0747318937,-1.3760257607	N,0,-0.0631401421,-1.0948921975,-1.2223939222
H,0,-2.0598549824,-3.8140149281,0.7330768645	H,0,-0.6898815044,-0.4834585564,-1.7554771633
H,0,-3.7097351108,-3.3187329862,0.2457492562	N,0,1.227018704,-0.644182905,-1.0515858297
H,0,-3.616709929,-2.656648023,2.6327709285	H,0,1.9702069851,-1.3234540879,-1.166575149
H,0,-1.9449173522,-2.0633894897,2.4163004234	C,0,1.4426526972,0.4288261308,-0.2270896428
H,0,-4.5358209538,-0.8606721093,1.3256784239	O,0,0.5224455831,1.1247863904,0.1711388946
H,0,-3.1915094923,-0.014062318,2.1359446925	C,0,2.8738083022,0.7645701769,0.1100184803
N,0,-2.7950465882,-0.649237055,0.1383189538	C,0,4.007123343,-0.042726606,-0.1288729228
C,0,-0.6437322086,-1.8056433087,-0.2007382662	C,0,3.0589872415,1.9957813249,0.7439753179
C,0,-2.8459908642,0.4139365645,-0.7149310887	C,0,5.2739538513,0.400320272,0.263874393
O,0,-2.3710635978,0.2968101296,-1.8480142888	C,0,4.3167595169,2.4429743537,1.1317263395
O,0,-0.0075454314,-2.323510138,0.696245068	H,0,2.1642792429,2.5911216479,0.9269652678
C,0,-3.4278012901,1.7734286849,-0.2707289718	C,0,5.423593357,1.6371719075,0.8873080821
C,0,-3.4011965651,2.6981524182,-1.4967141794	H,0,6.1544542425,-0.2149388675,0.0870603395
H,0,-4.032310046,2.3061685707,-2.3070471096	H,0,4.4302075108,3.4102565636,1.6220163876
H,0,-3.7759765278,3.6928490757,-1.2094960384	H,0,6.4219408999,1.9643822295,1.1835988293
H,0,-2.3824098606,2.8030118493,-1.8905364774	O,0,3.8136283608,-1.244926577,-0.7386290031
C,0,-2.5120517349,2.3750236019,0.8117836736	C,0,4.9138799001,-2.1142894739,-0.9160051276
H,0,-1.4670268383,2.3904923262,0.4728750996	H,0,5.6729347249,-1.6728851635,-1.582466798
H,0,-2.8357330881,3.4049319713,1.0308376121	H,0,5.375986872,-2.3771863557,0.0493178535
H,0,-2.5434391723,1.8122423125,1.7552169809	H,0,4.5079623411,-3.0200210694,-1.3807408418

Table S2. Cartesian coordinates for the optimized geometry of **S2**.

*Predicted CD spectrum of oligoindole (R,R)-**6***

To confirm that (*R*,*R*)-**6** does indeed fold into the *M* helix (which was not established experimentally in the original paper), model oligomer **S3** (Chart S3) was optimized in its helical conformation at the PCM(CH_2Cl_2)/ωB97-XD/cc-pVDZ level and its CD spectrum predicted using TD-DFT at the same level of theory. Optimization yields a geometry of energy −2141.76094367 E_h with no imaginary vibrational frequencies (C_1 symmetry). The geometry is shown in Figure S3 and the Cartesian coordinates in Table S3. The predicted CD spectrum is in Figure S4. We focus on the negative Cotton effect at roughly 320 nm, which is due to the conjugated backbone. This matches the long-wavelength Cotton effect observed in the experimental spectrum of (*R*,*R*)-**6**.[2]

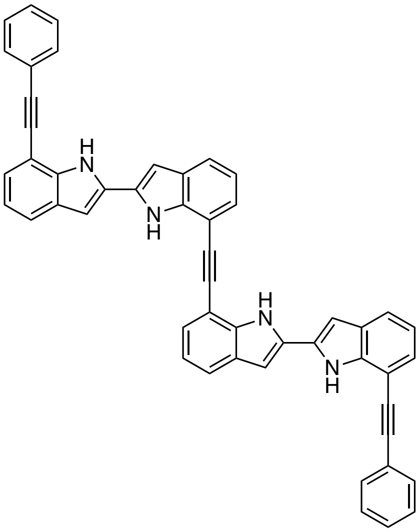

Chart S3. Oligoindole model structure **S3**.

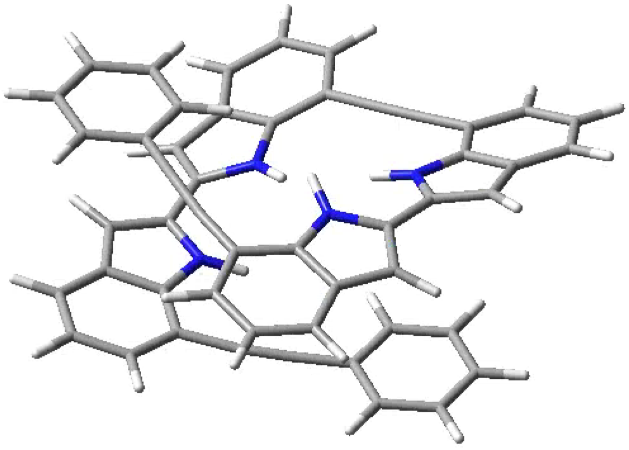

Figure S3. Optimized geometry of **S3**.

C,0,-0.4898606456,2.7618285673,-3.0738309103
C,0,-0.6550838494,4.1688298424,-3.0745747112
C,0,-0.5490603689,4.8658802853,-4.290297837
C,0,-0.2801936329,4.1532278487,-5.4511212263
C,0,-0.1173025995,2.757092432,-5.4334504871
C,0,-0.2205123984,2.0272122877,-4.2449370401
C,0,-0.9146281354,4.555414817,-1.7169379403
H,0,-0.6748655551,5.9495573109,-4.31877233
H,0,-0.1925657453,4.6811738607,-6.4018583759
H,0,0.0929045192,2.2228752472,-6.3607029791
H,0,-1.1034955814,5.5574192186,-1.3429393623
N,0,-0.6480804343,2.3251674977,-1.7862367577
H,0,-0.4927955553,1.3750730242,-1.4811698536
C,0,-0.891172406,3.4089807802,-0.9595909245
C,0,-1.0202495047,3.2268499347,0.4750371564
C,0,-0.7695881259,4.1060725126,1.5007551167
N,0,-1.3222969853,1.9826190255,1.0031836747
C,0,-0.9250908874,3.3759346733,2.7272272619
H,0,-0.424814897,5.1292112938,1.3846216252
C,0,-1.2852663885,2.0579596178,2.3688634618
H,0,-1.7400088453,1.2116939754,0.4989571337
C,0,-0.75364266,3.6780479105,4.0895385286
C,0,-1.5079012232,1.0374238105,3.3109028622
C,0,-0.9589474092,2.6768453102,5.030543515
H,0,-0.457424381,4.6811380896,4.4015514453
C,0,-1.3360747729,1.3740826053,4.6538344083
H,0,-0.8255198527,2.8990729863,6.0905184456
H,0,-1.4841940437,0.607279107,5.4149363318
C,0,-0.0685005777,0.6041089093,-4.2127545126
C,0,0.0566745329,-0.6073630743,-4.2124753298
C,0,0.2087268694,-2.0304789499,-4.2439394846
C,0,0.4813205124,-2.7641858215,-3.0730087275
C,0,0.1023457635,-2.7612694101,-5.4316173974
N,0,0.6429935422,-2.3265671147,-1.7861600187
C,0,0.6467237668,-4.1711675488,-3.0731770714
C,0,0.265373971,-4.1573946604,-5.4487016229
H,0,-0.1104674545,-2.2277665445,-6.3586866468
C,0,0.8884431341,-3.4097734489,-0.9593927812
H,0,0.4880873626,-1.3763538359,-1.4812571745
C,0,0.9100365461,-4.556738453,-1.7159766536
C,0,0.5375095483,-4.8691411835,-4.2880854792
H,0,0.1752300991,-4.6860559464,-6.398805453
C,0,1.0213819769,-3.2265674692,0.4747488685
H,0,1.1000883712,-5.558446039,-1.3417817429
H,0,0.6634019578,-5.9528203428,-4.3160866424
C,0,0.7735089577,-4.1049949078,1.5018238397
N,0,1.3248996758,-1.9819377139,1.0011033767
C,0,0.9323554309,-3.3739047178,2.7273016182
H,0,0.4284101043,-5.1282191964,1.3874222318
C,0,1.2916039922,-2.0562228493,2.3669356856
H,0,1.7412505099,-1.2114331876,0.4951155074
C,0,0.7645719759,-3.6749337364,4.0903092446
C,0,1.5168007193,-1.0349437066,3.307564653
C,0,0.9724584595,-2.6730001613,5.0299667404
H,0,0.4691593445,-4.6777654729,4.40391351
C,0,1.3486005231,-1.3705434786,4.6512176336
C,0,1.8487545323,0.2681220764,2.8260039978
H,0,0.8418938452,-2.8943934199,6.0904727525
H,0,1.4987861666,-0.6031457384,5.411315181
C,0,2.0915331901,1.3278638291,2.2801383346
C,0,-1.8411947378,-0.2660263496,2.8313055932
C,0,-2.0855276757,-1.3261752522,2.2869260663
C,0,2.3267309205,2.5690566546,1.6050502107
C,0,2.5721749,3.7486847811,2.3241039092
C,0,2.2732476733,2.617166022,0.2025862078
C,0,2.7475070383,4.9522337971,1.6478488737
H,0,2.6061987366,3.7148065597,3.4137445306
C,0,2.4430989092,3.8244706693,-0.466592376
H,0,2.0773971747,1.7027182697,-0.3592547828
C,0,2.6783218507,4.9953592411,0.2541430514
H,0,2.9325875539,5.8667494244,2.2140379564
H,0,2.3770446153,3.8525525097,-1.5557099693
H,0,2.8063696814,5.943552,-0.270761497
C,0,-2.3226446782,-2.5679043719,1.6134954401
C,0,-2.2730237462,-2.617165867,0.2109319133
C,0,-2.5661586286,-3.7469363752,2.3341818498
C,0,-2.4447715422,-3.8250099302,-0.4567922246
H,0,-2.0786890416,-1.703192674,-0.3522058087
C,0,-2.7433965992,-4.951031017,1.659397741
H,0,-2.5971866207,-3.7121680082,3.4238839083
C,0,-2.6780509388,-4.995302066,0.2655410518
H,0,-2.3817236645,-3.8539727712,-1.5460650889
H,0,-2.9269536477,-5.8650746903,2.2268436698
H,0,-2.807582517,-5.9439211144,-0.2582272287

Table S3. Cartesian coordinates for the optimized geometry of **S3**.

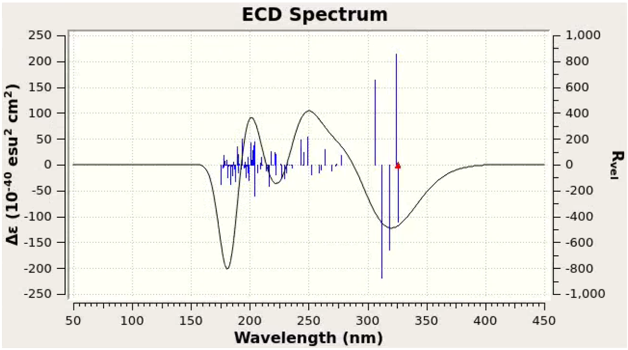

Figure S4. Predicted CD spectrum of **S3**.

*Analysis of oxymethylene (S,S)-**11***

A potential energy surface was generated for rotation about the indicated bond in **S4** (Chart S4) at the ωB97-XD/cc-pVDZ level. The PES is shown in Figure S5. It confirms that the *tert*-butyl group is antiperiplanar to the oxygen atom in the most favorable conformation.

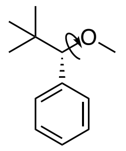

Chart S4. Oxymethylene terminus model structure S4.

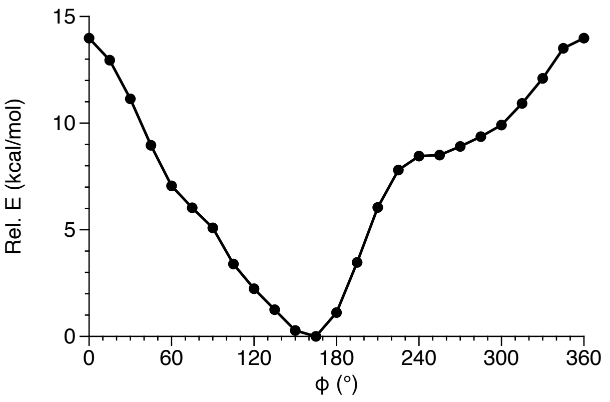

Figure S5. Potential energy surface for rotation about the key bond in S4.

References

(1) Frisch, M. J.; Trucks, G. W.; Schlegel, H. B.; Scuseria, G. E.; Robb, M. A.; Cheeseman, J. R.; Scalmani, G.; Barone, V.; Petersson, G. A.; Nakatsuji, H.; Li, X.; Caricato, M.; Marenich, A. V.; Bloino, J.; Janesko, B. G.; Gomperts, R.; Mennucci, B.; Hratchian, H. P.; Ortiz, J. V.; Izmaylov, A. F.; Sonnenberg, J. L.; Williams-Young, D.; Ding, F.; Lipparini, F.; Egidi, F.; Goings, J.; Peng, B.; Petrone, A.; Henderson, T.; Ranasinghe, D.; Zakrzewski, V. G.; Gao, J.; Rega, N.; Zheng, G.; Liang, W.; Hada, M.; Ehara, M.; Toyota, K.; Fukuda, R.; Hasegawa, J.; Ishida, M.; Nakajima, T.; Honda, Y.; Kitao, O.; Nakai, H.; Vreven, T.; Throssell, K.; Montgomery Jr., J. A.; Peralta, J. E.; Ogliaro, F.; Bearpark, M. J.; Heyd, J. J.; Brothers, E. N.; Kudin, K. N.; Staroverov, V. N.; Keith, T. A.; Kobayashi, R.; Normand, J.; Raghavachari, K.; Rendell, A. P.; Burant, J. C.; Iyengar, S. S.; Tomasi, J.; Cossi, M.; Millam, J. M.; Klene, M.; Adamo, C.; Cammi, R.; Ochterski, J. W.; Martin, R. L.; Morokuma, K.; Farkas, O.; Foresman, J. B.; Fox, D. J., *Gaussian 16, Rev. B.01*; Gaussian, Inc.: Wallingford, CT, 2017.

(2) Naidu, V. R.; Kim, M. C.; Suk, J.-m.; Kim, H.-J.; Lee, M.; Sim, E.; Jeong, K.-S. *Org. Lett.* **2008**, *10*, 5373–5376.
